# Health Tract No. 341

**Published:** 1868-07

**Authors:** 


HEALTH TRACT, NO. 341.

### WARMING HOUSES

Narrows itself down every year to the more universal use of coal; to the comparatively wealthy few, the use of coiled pipes conveying heated air or water, these pipes being so arranged that cold fresh air shall come into the room over, through and about them, will answer all the purposes of comfortable warmth and healthful ventilation. But the masses, in the present state of our knowledge, must burn coal in a furnace, to give out heat into the rooms through registers; in grates, in stoves or in fireplaces on a level with the floor, as in Dixon's Low Down Grate so commonly used in Philadelphia. This, whether peat, wood, soft or hard coal are used, is the cleanliest, least troublesome, most cheerful and healthful fire that can be made, to warm single rooms, costing from twenty-five to several hundred dollars each, according to finish. The cheapest method of warming a room with coal is by a stove, especially if the pipe is within seven feet of the floor; the greater its length, the cheaper the heat; and if so arranged, that the pure fresh air from without shall pour in against the bottom, top or back of the stove, and ventilating orifices be had at the most proper point, believed now to be in the floor, where the worst air is found, to be conveyed into the flue or chimney in contact with the under surface of the floor, which thereby is warmed more or less; this perhaps is the most comfortable, healthful and economical method of stove heat.

Grate heat is cheerful, cheap and dirty, and the most available mode of ventilation for the masses is to keep the door always wide open. But as our houses are already built with furnaces, to convey heat into the apartments through registers, our present knowledge seems to indicate that there should be an open fireplace in each room, or an aperture in the floor to let out the bad, heavy, used air, while a supply of fresh and pure air should come in through an open hall door, or its transom. But in all cases, of warming rooms, let the fireplace be always open; stop up no crevice with rags or cotton and away with all " weather strips," they keep out the cold, but they let in disease and death. In all cases of warming rooms, as our houses are at present constructed the most direct and the most easily understood method is to keep the door and fireplace always open and pile on the coal, a little at a time, to make up the difference, so that each one should have his room comfortably warm for himself and not be bothered with hygrometers, barometers, thermometers and gases oxide, carbonic, sulphuric mephitic and so on through the whole chemical catalogue.

